# Understanding the structural, optical, and dielectric characteristics of SrLaLiTe_1−*x*_Mn_*x*_O_6_ perovskites

**DOI:** 10.1038/s41598-021-89132-4

**Published:** 2021-05-07

**Authors:** M. Z. M. Halizan, Z. Mohamed, A. K. Yahya

**Affiliations:** Faculty of Applied Sciences, Universiti Teknologi MARA, 40450 Shah Alam, , Selangor Malaysia

**Keywords:** Condensed-matter physics, Materials for devices

## Abstract

In electronic applications, good dielectric permittivity material has huge potential in the capacitive energy storage devices. Herein, in the present work the dielectric study of SrLaLiTe_1−*x*_Mn_*x*_O_6_ (*x* = 0.02, 0.04, 0.06, 0.08, and 0.10) double perovskites has been studied and discussed. These compounds were prepared through solid-state reaction method. All of the prepared compounds were confirmed to crystallized in monoclinic structure of *P2*_*1*_*/n* space symmetry with better crystallization when dopant concentrations increased until *x* = 0.08. The formation of Li–O–Te/Mn bonds in octahedral structures in all compounds were confirmed in this study. The existence of peaks at specific wavenumbers indicated vibrations of B–site cations’ bonds. When dopant amounts were increased from* x* = 0.02 to *x* = 0.08, there was an increasing trend of grains sizes formation in the compounds. The discussions on effects of grain sizes towards dielectric properties were included in this paper. Other important results and discussions comprised of the significant effects of dopant on the optical band gap (*E*_*opt*_) and absorption frequencies of the compounds. The decreasing trend of *E*_*opt*_ towards semiconductor range indicated the compounds’ promising potentials for optoelectronic device application.

## Introduction

Research and studies regarding perovskites have been instigated extensively due to its promising abilities such as superconducting, conductivity, magnetoresistance and ferroelectric. Pertaining to these astounding characteristics, they are growing appeals for applications in sensors^1–3^, capacitors^4^, microwave resonators^5–7^, and solar cells^8–11^. Double perovskites oxides established an ordered rock–salt–like structure of BO_6_ and B'O_6_ units in the crystal. They are basically comprised of *A*_*2*_*BB*'*O*_*6*_ or *AA*'*BB*'*O*_*6*_ configuration where A or A' are alkaline–earth or rare–earth metals in Group I or Group II while B and B' are transition metals. These configurations were derived from conventional *ABO*_*3*_ perovskites where six out of twelve A–site cations and six B–site cations replaced with A'– and B'–sites cations, respectively. As double perovskites have more sites for substitutions with additional A'– or B'–sites compared to conventional perovskites, they can have substantial advantages for instance higher Curie temperature (*T*_*C*_) such as Sr_2_CrReO_6_ and Ba_2_FeMoO_6_ with *T*_*C*_ above 300 K^12,13^ compared to perovskites like RCu_3_Mn_4_O_12_ and Sr_0.9_Sn_0.1_TiO_3_ that possesses *T*_*C*_ of 50 K and 200 K, respectively^14,15^. The field has gradually broadened as tellurium based double perovskites, *AA*'*BTeO*_*6*_ has been studied and reported to have relatively good dielectric properties^16–19^. Variety of discussions which consisted of densification, grain size or polarizability aspects of A–site cations has been included in these studies. Double perovskites with *AA*'*BB*'*O*_6_ form that have 1:1 B-site ordering has been reported to have potential in applications of dielectric^19^. Furthermore, double perovskites in *AA*'*BB*'*O*_6_ configuration and form polar *P2*_*1*_ space group symmetry can be related to existence of ferroelectric or good dielectric properties^20^. Since SrLaLiTeO_6_ claimed to consist of the 1:1 B site ordering of Te^6+^/Li^+^ and monoclinic *P2*_*1*_*/n* structure^21^ that is similar to that in the SrBiLiTeO_6_, BaBiLiTeO_6_, BaBiNaTeO_6_, and BaLaNaTeO_6_^18,19^, it could be suggested that SrLaLiTeO_6_ has good dielectric abilities.

Apart from that, the properties of double perovskites materials mainly depend on B–site cations arrangements which able to modify the electrical or magnetic properties. The position of B– and B'–cations in octahedral are alternating if the size difference is large meanwhile random placement of the B– and B'–cations take place whenever the size difference is small. Study regarding Ba_2_ZnWO_6_ double perovskite in microwave frequencies points out that small dope of insulator and larger size cation (Ca^2+^) into B–site of perovskite could affect its microwave dielectric properties^22^ by altering the tolerance factor of compound. The open problem in our study is regarding the ability of B–site cations doping to alter the dielectric properties of Te–based double perovskite for dielectric application such as capacitor or resonator. Studies by Vilesh et al. regarding BaBiNaTeO_6_ and BaBiLiTeO_6_^18,19^ indicated that different B–site doping can influence dielectric constant and dielectric loss. However, there is no direct study regarding this B–site full cations difference and its effects towards compounds’ dielectric properties. Nonetheless, since Na^+^ and Li^+^ has almost same valence electron configuration, the main difference could be their ionic size and hence, altering cations bond stress and strain to cause different structural (octahedral) tilting or distortion before affecting other aspects such as formation energy^23^. Thus, the idea in this study is to alter the octahedral tilting or distortion in Te–based double perovskite through B–site cations doping with smaller cations to tune the structure of compounds and its dielectric property accordingly.

Meanwhile, study on B–site doping in Ba_2_ZnWO_6_ has been conducted^24^ and demonstrated B–site cations doping with electrically conductive characteristic has potential to yield difference on optical property of double perovskite compound. Cations with good conductivity which doped into B–site is vital in controlling charge carrier’s movement or Fermi energy level inside perovskite’s lattice structure, hence, reducing optical band gap (*E*_*opt*_). This will take effect simultaneously with the effect of structural distortion onto optical band gap since distortion can affect the band gap^21^. By doping smaller cation with electrically conductive trait into B–site of SrLaLiTeO_6_, the dominant effect whether reducing or widening of its *E*_*opt*_ can be seen.

There are some reports that showed enhancement of dielectric constant and narrowing of the optical band gap simultaneously^25,26^. Therefore, investigating the effects of B–site cation doping in SrLaLiTeO_6_ with Mn^6+^ (0.255 Å) on its structural, dielectric and optical properties is an interesting endeavour and this work would deal to ascertain these effects.

## Materials and methods

The SrLaLiTe_1−*x*_Mn_*x*_O_6_ (*x* = 0.02, 0.04, 0.06, 0.08, and 0.10) compounds were prepared by solid-state reaction method^21^. High purity chemicals (≥ 99.99%) of strontium carbonate (SrCO_3_), lanthanum oxide (La_2_O_3_), lithium carbonate (Li_2_CO_3_)_,_ tellurium dioxide (TeO_2_), and manganese (III) oxide (Mn_2_O_3_) powders were purchased from Sigma-Aldrich. The chemicals were mixed at required stoichiometric ratios before grinded with pestle and agate mortar in 1 h duration to attain homogeneity. The mixed powders were calcinated in air using a CWF 11/5 furnace (Carbolite Gero, UK) with heating rate of 15 °C/min until max. temperature of 850 °C in 10 h period. Then, slow cooling rate of 1 °C/min was done in order to ensure the formed stoichiometry match with the desired oxygen stoichiometry^27^. The samples then were pressed to form pellet at pressure of 5 T by using Atlas 15 T hydraulic press (Specac, UK) before sintered in air at max. temperature of 850 °C in 10 h duration. The phase(s) and purity of the samples were investigated by the x–ray diffraction (XRD) characterization within the angle range of 10° to 80° using Xpert PRO MPD diffractometer (PANanalytical, Netherland). The instrument equipped with a Cu K_α_ radiation which has 1.5418 Å of wavelength. The graphical user interface (EXPGUI) and visualisation for electronic structural analysis (VESTA) software were deployed for structural refinement and to visualize the refined structure of compounds, respectively^18,28–30^. The infrared absorption spectra of the samples were collected by Fourier transform infrared (FTIR) characterization within the wavenumber range of 400 to 1500 cm^−1^ using Drift Nicolet 6700 spectrometer (Thermo Fisher, USA). The morphologies and constituents in the samples were determined by field emission scanning electron microscope (FESEM) and energy dispersive x–ray (EDX) characterizations, which were conducted using SU 8000 FESEM (Hitachi, Japan). The dielectric characteristics of the samples were obtained by electrochemical impedance spectroscopy (EIS) characterization within the frequency range of 50 Hz to 1 MHz and temperature range of 298 K to 343 K using LCR 3532–50 HiTester analyzer (Hioki, Japan) while keeping the compounds and electrodes in sandwich geometry. The optical spectra of the samples were investigated by ultraviolet–visible light (UV–vis) characterization within the wavelength range of 200 to 1000 nm using Lambda 750 spectrometer (Perkin Elmer, USA). The densities of each samples were calculated by applying the Archimedes’ formula.

## Results and discussions

**Figure 1:**
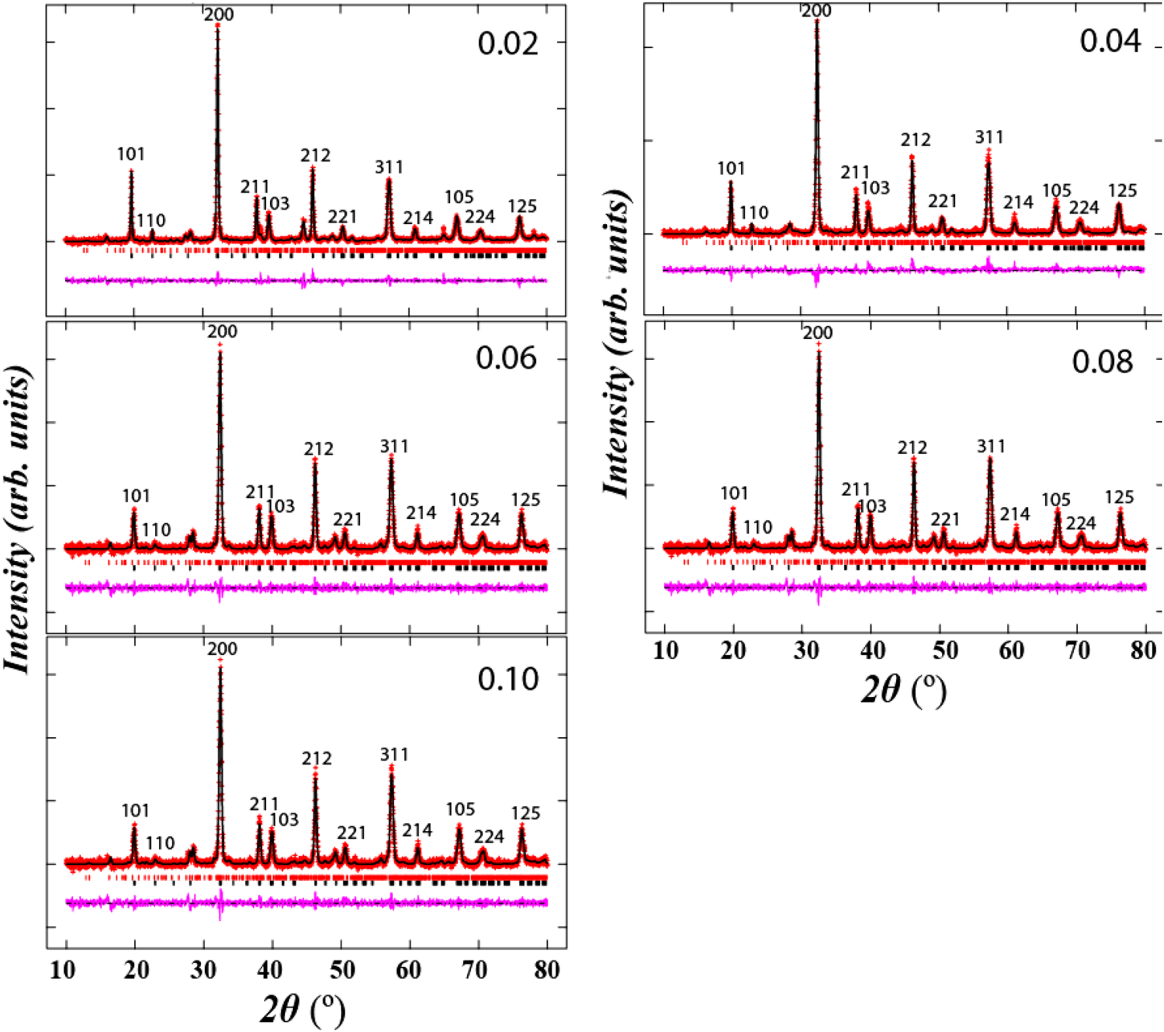
Refinement of XRD pattern of SrLaLiTe_1−*x*_Mn_*x*_O_6_. The black lines, red lines and pink lines are the calculated pattern, the observed data, and the difference, respectively. Black and red ticks are the Bragg reflections for primary and secondary phases, respectively.

**Figure 2:**
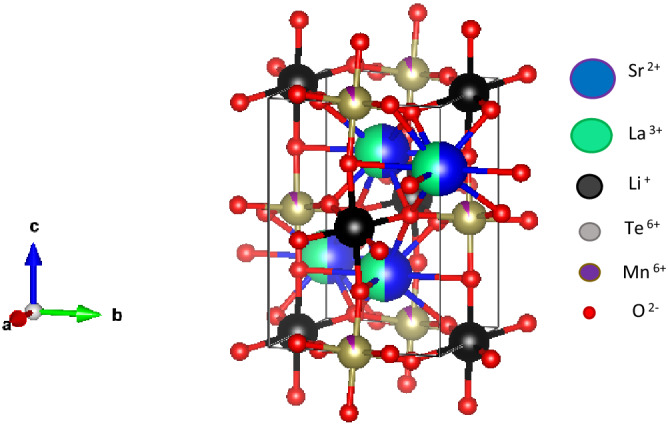
Image of refined XRD in SrLaLiTe_1−*x*_Mn_*x*_O_6_ from *bc* plane.

Figure 1 shows the refined XRD data of SrLaLiTe_1−x_Mn_x_O_6_ (*x* = 0.02 (Mn 0.02), *x* = 0.04 (Mn 0.04), *x* = 0.06 (Mn 0.06), *x* = 0.08 (Mn 0.08), and *x* = 0.10 (Mn 0.10)) by the Rietveld refinement method. From these plots, all compounds were formed in double phase, with minor peaks present corresponded to Sr_7_Mn_4_O_12_ with percentage of less than 10%. The obtained reliabilities (*χ*^*2*^) were 2.178, 1.903, 1.647, 2.419, and 1.948 for compounds with* x* = 0.02, 0.04, 0.06, 0.08, and 0.10, respectively which showed good reliability of results. All prepared compounds were crystallized in monoclinic structure of *P2*_*1*_*/n* symmetry. The refined lattice parameters of *a*, *b*, and* c* in all compounds were in 5.57–5.63 Ǻ, 5.58–5.61 Ǻ, and 7.1–7.92 Ǻ ranges, respectively. All compounds possessed *α* = 90° and *γ* = 90°, whereas *ß* = 90.37°, 90.25°, 89.93°, 90.05°, and 90.10° for *x* = 0.02, 0.04, 0.06, 0.08, and 0.10, respectively. The obtained unit cell volumes (*V*) for all compounds were 248.7, 247.7, 248.5, 249.5, and 249.9 Å^3^, respectively. The trend was not in agreement with the doping of smaller size of Mn^6+^ into larger Te^6+^ cations. Table 1 shows the complete parameters that were obtained from the refinement. Figure 2 shows refined structure of SrLaLiTe_1−*x*_Mn_*x*_O_6_ from *bc* plane. This figure revealed presence of doped Mn^6+^ into Te^6+^ alongside Li^+^ at B–site octahedral structure. Mn^6+^/Te^6+^ and Li^+^ alternately positioned between each other and being surrounded by six O^2−^ atoms in each octahedron. A-site cations (Sr^2+^ and La^3+^) placed between octahedral to fill up spaces in the structure. In all compounds, Li^+^ was located at (0.5, 0, 0) and Te^6+^ was detected at (0, 0.5, 0) coordinates. The tolerance factor (*τ*) of the compounds was calculated by the equation below^21^:

1 $$\tau = \frac{{\frac{{R_{a} + R_{{a^{{\prime }} }} }}{2} + R_{o} }}{{\sqrt 2 \left( { \frac{{R_{b} + R_{{b^{{\prime }} }} }}{2} + R_{o } } \right)}}$$

where R_a_ and R_a'_ are radii of A–site cations (Sr^2+^ and La^3+^), R_b_ and R_b'_ are radii of B-site cations (Li^+^ and Te^6+^/Mn^6+^) and R_o_ is radius of oxygen anion (O^2−^). Size of ionic radius used were 1.44 Å (Sr^2+^) and 1.36 Å (La^3+^) with CN:12 while 0.76 Å (Li^+^), 0.56 Å (Te^6+^), 0.255 Å (Mn^6+^), and 1.40 Å (O^2−^) with CN:6^31^. The evaluated *τ* are presented in Table 1. The increment of *τ* towards value of 1 (the ideal cubic structure) showed the positive effect of doping Mn^6+^ in reducing distortion in perovskite structure. The increment indicated that the doping of smaller ionic into B–site does aid in layered formation of A–site. Besides, the value of octahedra tilting angle (*ϕ*) in each compound were calculated by using equation:

2 $$\phi = \frac{180 - \theta }{2}$$

where *θ* is average angles for (Li–O–Te/Mn) bond^32^. All compounds possessed the same tilting angle which were 9.6°. This indicates that tilting angle cannot be affected by small doping into pristine SrLaLiTeO_6_. The average Li–O– Te/Mn bond angle in each compound are the same and in agreement with unaffected tilting angle of octahedral structures. Length of each (B–O) and (A–O) bonds showed increasing trend as doping concentration enhanced. The increase most probably due to the smaller ionic radius doping and for that reason, causing each bond in A– and B–sites to elongate to accommodate the unaffected tilting of octahedrons. The crystallite size (*D*) was determined by applying the Scherrer formula^33^:

3 $$D = \frac{K\lambda }{{\beta \left( \theta \right)\cos \theta }}$$

where *K* is constant value, *β* is the full width at half maximum (FWHM), *θ* is the angle of XRD peaks, and *λ* is the wavelength of XRD beam. The calculated *D* for each compound were 28.99, 20.19, 23.48, 26.84, and 26.41 nm for Mn 0.02, Mn 0.04, Mn 0.06, Mn 0.08, and Mn 0.10, respectively. Increasing trend of *D* from Mn 0.04 to Mn 0.08 indicates that Mn^6+^ dopant resulted the better crystallization as doping contents increased.

**Table 1:** Obtained space symmetry, parameters of lattice, *V*, angles of bonds, lengths of bond, and fit goodness from Rietveld refinement with calculated *ϕ*, *τ*, and *D* in SrLaLiTe_1−x_Mn_x_O_6_.

Compounds	Mn 0.02	Mn 0.04	Mn 0.06	Mn 0.08	Mn 0.10
**Lattice parameter**					
Space symmetry	*P2*_*1*_*/n*	*P2*_*1*_*/n*	*P2*_*1*_*/n*	*P2*_*1*_*/n*	*P2*_*1*_*/n*
*a* (Å)	5.598(8)	5.578(10)	5.621(4)	5.628(2)	5.595(2)
*b* (Å)	5.607(1)	5.614(8)	5.582(4)	5.591(8)	5.630(2)
*c* (Å)	7.926(5)	7.911(16)	7.919(6)	7.923(3)	7.933(2)
*α*	90.00°	90.00°	90.00°	90.00°	90.00°
*ß*	90.37°	90.25°	89.93°	90.05°	90.10°
*γ*	90.00°	90.00°	90.00°	90.00°	90.00°
Unit cell Vol., *V* (Å^3^)	248.7	247.7	248.5	249.5	249.9
**Bond length (Å)**					
Li-O_1_ (× 2)	2.123(6)	2.119(5)	2.122(4)	2.126(5)	2.125(7)
Li-O_2_ (× 2)	2.085(6)	2.081(5)	2.086(4)	2.089(4)	2.087(8)
Li-O_3_ (× 2)	2.064(11)	2.060(8)	2.063(6)	2.065(6)	2.067(9)
Aver. < Li–O >	2.091(8)	2.087(6)	2.090(5)	2.093(5)	2.093(8)
Te-O_1_ (× 2)	1.934(5)	1.933(4)	1.935(4)	1.937(4)	1.940(7)
Te-O_2_ (× 2)	1.936(5)	1.935(4)	1.934(3)	1.937(3)	1.941(7)
Te-O_3_ (× 2)	1.924(10)	1.920(8)	1.921(6)	1.924(5)	1.925(8)
Aver. < Te–O >	1.931(7)	1.929(5)	1.930(4)	1.933(4)	1.935(7)
Mn-O_1_ (× 2)	1.934(5)	1.933(4)	1.935(4)	1.937(4)	1.940(7)
Mn-O_2_ (× 2)	1.936(5)	1.935(4)	1.934(3)	1.937(3)	1.941(7)
Mn-O_3_ (× 2)	1.924(10)	1.920(8)	1.921(6)	1.924(5)	1.925(8)
Aver. < Mn–O >	1.931(7)	1.929(5)	1.930(4)	1.933(4)	1.935(7)
Aver. < Sr-Sr >	3.962(19)	3.958(18)	3.962(8)	3.965(11)	3.969(24)
Aver. < La-La >	3.962(19)	3.958(18)	3.962(8)	3.965(11)	3.969(24)
**Bond angles (°)**					
Li-O_1_-Te/Mn	155.0(12)	155.0(10)	155.1(9)	155.1(9)	155.1(12)
Li-O_2_-Te/Mn	160.2(4)	160.2(4)	160.3(6)	160.3(8)	160.3(5)
Li-O_3_-Te/Mn	167.1(8)	167.1(6)	167.1(4)	167.1(4)	167.1(7)
Aver. < Li–O-Te/Mn >	160.7(8)	160.7(7)	160.8(6)	160.8(7)	160.8(8)
**Fit goodness**					
*χ*^2^	2.178	1.903	1.647	2.419	1.948
R_*p*_ (%)	0.117	0.133	0.138	0.135	0.119
R_*wp*_ (%)	0.167	0.171	0.179	0.183	0.158
**Tolerance factor and tilting angle**					
Tol. factor, *τ*	0.963	0.964	0.965	0.967	0.968
Tilting angle, *Φ*	9.6	9.6	9.6	9.6	9.6
**Crystallite size (nm)**					
Crystallite Size, *D*	28.99	20.19	23.48	26.84	26.41

**Figure 3:**
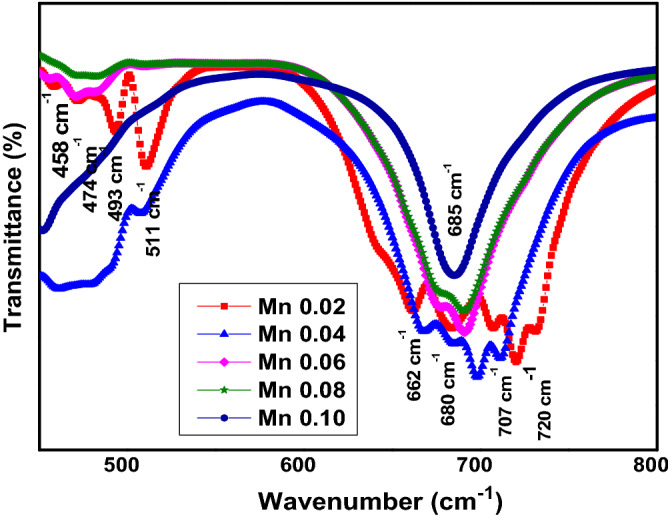
FTIR spectrum of SrLaLiTe_1−*x*_Mn_*x*_O_6_.

Figure 3 illustrates the FTIR spectrum of SrLaLiTe_1−*x*_Mn_*x*_O_6_ (*x* = 0.02, 0.04, 0.06, 0.08, and 0.10) compounds. As a comparison, Mn 0.02 presents significant peaks between 400 to 1000 cm^−1^. Peaks at 458 and 472 cm^−1^ in Mn 0.02 correlated with Li-O bond stretching vibrations in octahedral structures^34^. The emergence of medium peaks at 495 cm^−1^ can be detected and be assigned to antisymmetric stretching vibrations (*v*_1_) of Te–O bonds in the octahedral structures. At the same time, strong peaks at 662, 680, and 710 cm^−1^ were clear and can be related to the symmetric stretching vibration (*v*_2_) of the Te–O bonds^19,35–38^. It is apparent that as dopant concentration increased, there were more quenches of peaks. This is understandable since the Te–O–Li should be reduced when the doping Mn^6+^ into Te^6+^ took place to form Mn–O–Li bonds. Nonetheless, peak at 718 cm^−1^ in Mn 0.02 can be suggested due to introduction of Mn^6+^ to form Mn–O bonds with none of peak present at the same wavenumber for pristine SrLaLiTeO_6_^39^. Besides, this peak started to shift to lower wavenumbers indicates the increment of abundance of Mn–O bonds as dopant increased with Mn 0.10 depicts the Mn–O bonds vibration peak at 685 cm^−1^. Reports have claimed that the Mn–O bonds with inclusion of Mn^3+^ / Mn^4+^ showed vibration peaks at 600 cm^−1^ or above^40,41^. Since Mn^6+^ has smaller ionic radius compared to Te^6+^ and Mn^3+^ / Mn^4+^, hence, it is possible for Mn^6+^ that form Mn–O bonds to exhibit vibration peaks at higher wavenumbers. Meanwhile, most of the existing peaks redshifted as dopant content increases implying the increase of bond length. These results are in accordance with the Rietveld refinement and tolerance factor. Summary of the peaks obtained were tabulated in Table 2.

**Table 2:** Samples’ peaks and their assignments from FTIR characterization.

Compounds’ wavenumber peaks (cm^−1^)					Assignments
Mn 0.02	Mn 0.04	Mn 0.06	Mn 0.08	Mn 0.10	
458	460	457	–	–	Stretching Li–O
474	480	457	470	453	Stretching Li–O
493	491	482	483	481	*v*_1_
511	507	509	507	515	*v*_1_
662	–	–	–	–	*v*_2_
680	–	–	–	–	*v*_2_
707	684	678	678	–	*v*_2_
720	698	691	690	685	Stretching of Mn–O

**Figure 4:**
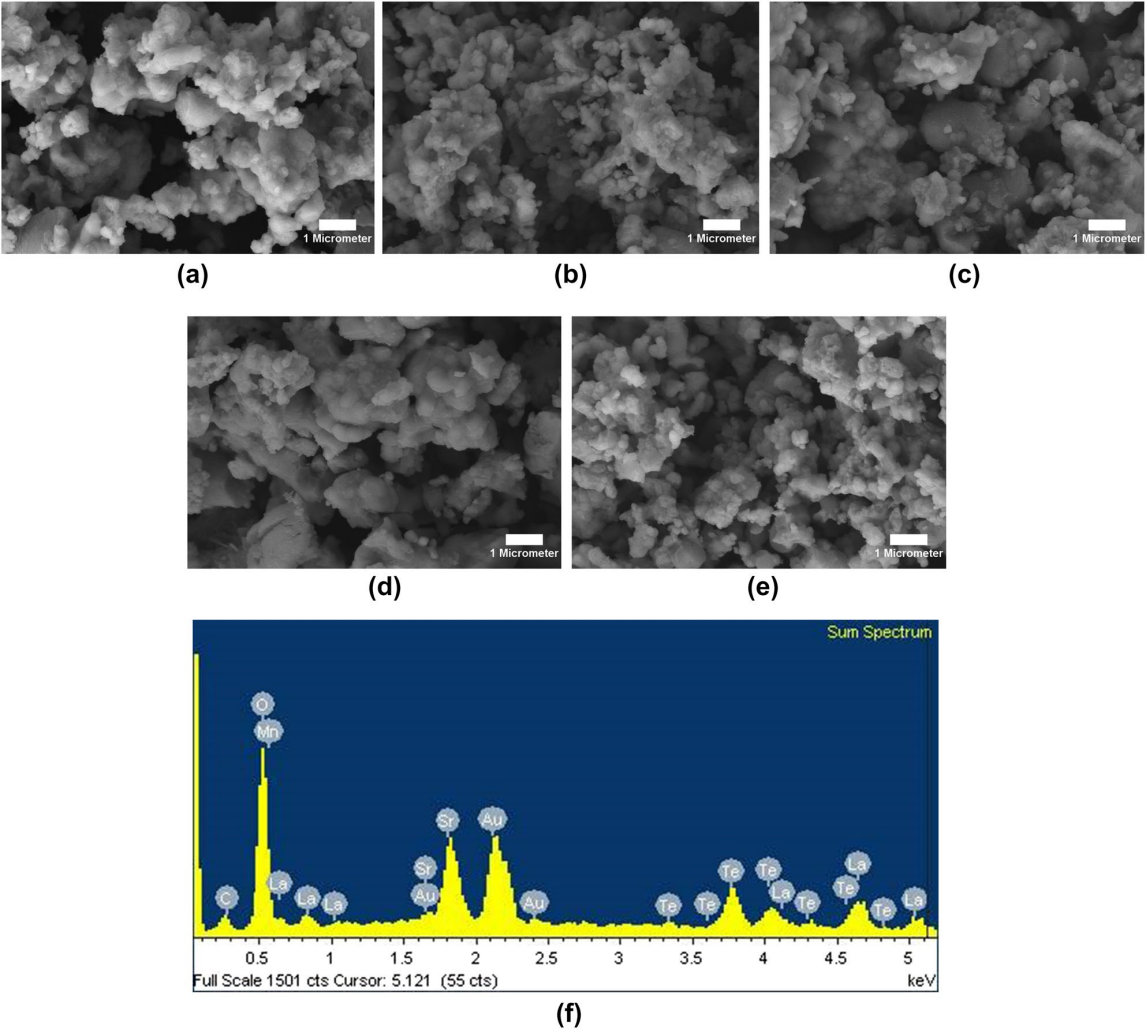
FESEM images (10 K magnification) of SrLaLiTe_1−*x*_Mn_*x*_O_6_ in powder form and EDX plot of *x* = 0.08.

Figure 4 exhibits the morphology of SrLaLiTe_1−*x*_Mn_*x*_O_6_ (*x* = 0.02, 0.04, 0.06, 0.08, and 0.10) compounds. The formation of agglomerated particles with same shape and size distribution of most grains were clear. The measured average grain sizes were increased from 1.03–1.97 µm, 1.10–1.17 µm, 1.97–2.07 µm, 2.14–2.26 µm and 1.21–1.43 µm as dopant concentration increased from *x* = 0.02 to *x* = 0.10. This variation trend was same with trend of crystallite size variation. The increasing trend of size of grain in Mn 0.04 to Mn 0.08 most probably related to decrease in structural distortion when smaller ionic radius (Mn^6+^) content increased^23^ and thus, affecting rate of nucleation^42^. Other possible reason may be due to the reduction of grain boundaries energy when Mn contents increased and lead to grain size enhancement. EDX graph in Figure 4f exhibits constituted elements in Mn 0.08. This graph confirms that this compounds contains elements of the prepared raw material composition, except Au which originate from coating in sample preparation for FESEM characterization. Other compounds show almost same trend of EDX graph as in Figure 4f. As calculated by Archimedes method, the density of all compounds had difference of less than 4%. The densest compound was Mn 0.06 with 5.450 g cm^−3^ despite Mn 0.08 having the largest grain sizes. This occurrence most likely because of less porosity in Mn 0.06 compared to all samples.

**Figure 5:**
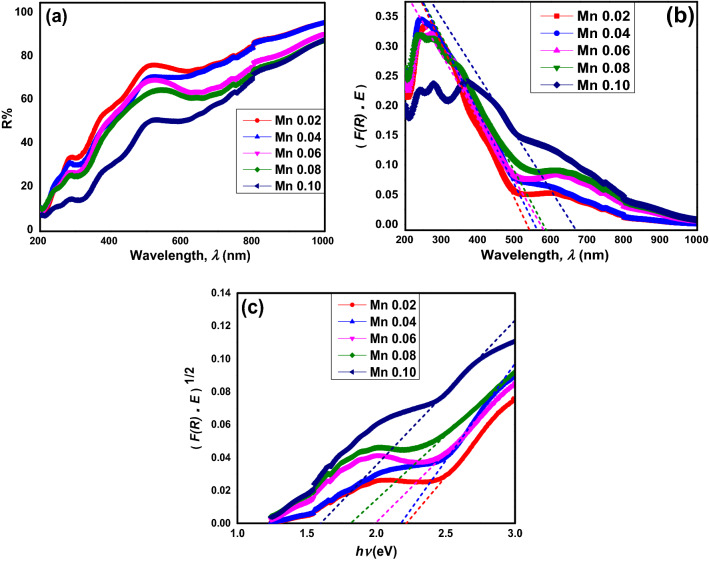
(**a**) The diffuse reflectance, (**b**) Kubelka–Munk, and (**c**) Tauc plotting of SrLaLiTe_1−*x*_Mn_*x*_O_6_ (dotted line are the fitting for optical band gap).

Figure 5a exhibits the UV–vis reflectance spectra versus wavelength. Compared to pristine SrLaLiTeO_6_^43^, there are strong absorption bands that were observed at the whole 300–800 nm range. It was probably because of the charge transfer between *3d* orbital of Mn^6+^ and *2p* orbital of O^2−^. As Mn^6+^ dopant increases, the reduction of reflectance value of compound indicates the increase of the absorbance character. Spectra for the compounds show the changes in shoulder in the spectrum just below 800 nm in comparison to pristine SrLaLiTeO_6_^43^, suggesting the *E*_*opt*_ of the doped compounds are dramatically altered. When Mn^6+^ doped into Te^6+^ site, Mn band could be formed within the gap. At low concentration of dopant, the band gap width is not hugely affected. With increasing Mn^6+^ concentration, the widths of Mn bands could increase and the overlapping of the bands could happen. Hence, implies the changes in the reflectance spectra^44^.

Figure 5b shows the absorption UV–vis of SrLaLiTe_1−x_Mn_x_O_6_ plotted from Kubelka–Munk equation^21^,

4 $$F\left( R \right) = \frac{{\left( {1 - R} \right)^{2} }}{2R}$$

where *R* is diffuse reflectance of compounds. The value of absorption edge can be determined by extrapolate the plot to zero absorption. Then, the *E*_*opt*_ values can be calculated by using formula:

5 $$E_{g} = \frac{1240}{\lambda }$$

where *λ* is the absorption edge’s wavelength. Figure 5c shows the graph plotted by using the Tauc equation:

6 $$\left[ {F\left( R \right)hv} \right]^{n} = A\left( {hv - E_{opt} } \right)$$

where *hv* is photon energy, *A* is proportional constant, and *E*_*opt*_ is gap energy. Meanwhile, the values of *n* can vary based on the of transition type in a material where *n* = 1/2 indicate the direct with allowed transition,* n* = 2 indicate the indirect with allowed transition,* n* = 3/2 indicate the direct with forbidden transition, and* n* = 3 indicate the indirect with forbidden transition^21^.

Figure 5b shows the absorbance cut–off wavelength increases with increasing Mn^6+^ content. Meanwhile, Fig. 5c shows fitting of *n* = 1/2 is the most fitted to the graph and hence, implies that only allowed and direct shift of electrons took place from highest occupied molecular orbital (HOMO) to lowest unoccupied molecular orbitals (LUMO) in these compounds where only photon absorption occurred. The comparable values of *E*_*opt*_ obtained from both graphs are tabulated in Table 3. *E*_*opt*_ values from Tauc plot are smaller than the ones from Kubelka–Munk calculations. Based on Tauc plot, all obtained *E*_*opt*_ values are in the range of 2.18 eV until 1.55 eV, indicating the semiconductor optical properties. The value getting smaller from Mn 0.02 until Mn 0.10. These values show much reduction of *E*_*opt*_ compared to pristine SrLaLiTeO_6_^21^. Literature^44,45^ has reported that *E*_*opt*_ is correlated to the existence of energy levels of impurities within the *E*_*opt*_ of materials. As wider Mn energy bands (impurity bands) formed with higher dopant concentration, this change reduced the distance between bands and consequently reduced the *E*_*opt*_. On the other hand, reduction in structural distortion when Mn^6+^ doped into pristine compound is the other reason to reduce *E*_*opt*_. Opposite explanation regarding effect of distortion onto band gap has been discussed in other report^21^. Besides, oxygen vacancies were the other possible factor which could promote the formation of energy levels of impurities within the *E*_*opt*_^45^ in all compounds. It is accepted that oxygen vacancies can affect the optical result. Nonetheless, lattice parameters as well as monoclinic structure in every compounds were close to the ones in SrLaLiTeO_6_ on previous report^21^. It is suggested that there were minor differences of oxygen content among the compounds. Therefore, oxygen vacancies should not show major effects towards the optical properties. There is other report that revealed the same trend of *E*_*opt*_ in other compound with doping of Mn^2+^ realized^46^. The absorbance spectra in Fig. 5a shows that all compounds’ optical band gaps are in visible light range, makes SrLaLiTe_1−*x*_Mn_*x*_O_6_ possible for photovoltaic applications with further study to increase the electrons or holes  mobility.

**Table 3:** Absorbed wavelength from Kubelka–Munk plot, *E*_*opt*_ from Kubelka–Munk plot, and *E*_*opt*_ from Tauc plot in SrLaLiTe_1−*x*_Mn_*x*_O_6_.

Compounds	Mn 0.02	Mn 0.04	Mn 0.06	Mn 0.08	Mn 0.10
*λ *_*abs*_ (nm)	544	556	565	578	664
*E*_*opt abs*_ (eV)	2.27	2.23	2.19	2.15	1.87
*E*_*opt Tauc*_ (eV)	2.18	2.14	1.95	1.77	1.55

**Figure 6:**
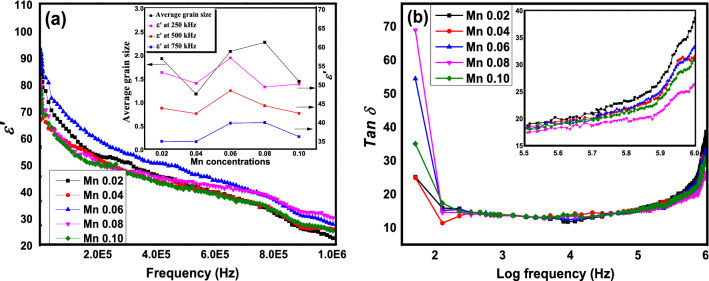
(**a**) Variation of $\varepsilon^{{\prime }}$ and (**b**) *Tan δ* of SrLaLiTe_1−*x*_Mn_*x*_O_6_, (inset of (**a**)) average grain sizes and $\varepsilon^{{\prime }}$ at specific frequencies for each compounds, (inset of (**b**)) *Tan δ* at 300 kHz and higher frequencies.

Figure 6a exhibits the plot of $\varepsilon^{{\prime }}$ with respect to frequencies from 50 Hz to 1 MHz in SrLaLiTe_1−*x*_Mn_*x*_O_6_ (*x* = 0.02, 0.04, 0.06, 0.08, and 0.10) compounds in room temperature. The variation of $\varepsilon^{{\prime }}$ with frequency showed almost the same trend for each compound, with respect to frequencies below or above 100 Hz. For frequencies below 100 Hz, all compounds showed almost similar variation of with frequency where $\varepsilon^{{\prime }}$ dropped instantaneously when frequency increased. At frequencies above 100 Hz, the decrease of $\varepsilon^{{\prime }}$ was moderately and steadily to reach the minimum value at 1 MHz. Compound Mn 0.06 showed the highest $\varepsilon^{{\prime }}$ values from 50 Hz to 700 kHz. However, started from 300 kHz, the gradient of decrease of $\varepsilon^{{\prime }}$ in Mn 0.08 was the most minimum among all compounds before showed the highest value of $\varepsilon^{{\prime }}$ at 1 MHz. Figure 6a (inset) shows changes of at 250 kHz, 500 kHz, and 750 kHz frequencies together with average size of grains for all compounds. It is clear that aside of $\varepsilon^{{\prime }}$ in Mn 0.02, the increasing trend of $\varepsilon^{{\prime }}$ observed with the peak value shown by Mn 0.08 before decrease significantly at higher doping concentrations. This $\varepsilon^{{\prime }}$ peak most probably interrelated with peak of grain size of Mn 0.08*.*

Figure 6b exhibits the dielectric loss, *Tan δ* variation in SrLaLiTe_1−*x*_Mn_*x*_O_6_ (*x* = 0.02, 0.04, 0.06, 0.08, and 0.10) with respect of frequencies in room temperature. This figure shows drop of *Tan δ* in all compounds at frequencies less than 500 Hz before relatively flat pattern at frequencies above 500 Hz. Compounds with different dopant concentrations exhibit almost the same increasing pattern from 10 kHz to 1 MHz. Figure 6b (inset) shows *Tan δ* at 300 kHz and above, where all compounds registered increasing *Tan δ* values at this range of frequencies.

The real part of dielectric constant, $\varepsilon^{{\prime }}$ illustrates the electrical dipoles’ aligning ability in compounds with external electric field. The high values of $\varepsilon^{{\prime }}$ at minimum frequencies in all compounds from Fig. 6a were suggested because of the space charge polarization that primarily consists of the accumulated heavy electrical dipoles at grain/grain boundaries interfaces when electrical field alternation existed. Besides, the minor possibility for oxygen vacancies existence could reduce the phonon modes in the compounds’ structure, hence, elicited the space charge polarization^47^. The decrease of $\varepsilon^{{\prime }}$ at low frequencies in all compounds was because of these heavy dipoles were impotent to move with the alternation of external field when frequencies were increased. $\varepsilon^{{\prime }}$ drop in low frequencies was in alignment with high losses in *Tan δ* at the same frequencies range as in Fig. 6b.

*Tan δ* illustrates the energy loss in the compounds in the midst of electric field alternation. The presence of peak at minimum frequency in Fig. 6b could be due to the impotency of heavy electrical dipoles to move with the external electric field or DC conduction loss. The primary reason for the loss could be the inability of electrical dipoles to move with the field changes as frequency increased. Another plausible reason is the charge carriers’ motions across the grain boundaries. Any minor possible formation of singly/doubly ionised oxygen vacancies that were due to the evaporation of lithium in the midst of sintering could produce charge carriers^48^ by the following reaction:

7 $$O_{o} \to \frac{1}{2} O_{2} + V_{o}^{{\prime \prime }} + 2e^{ - 1}$$

where $V_{o}^{{\prime \prime }}$ is double ionised oxygen vacancies. Hence, DC conduction loss might be another factor which assisted the drop of the $\varepsilon^{{\prime }}$. However, the gradient in plot of ln $\varepsilon^{{\prime }}$ against ln *ω* (not shown) did not show magnitude close to (-1) to prove the presence of this factor. Meanwhile, the increase of Tan δ starting from 300 kHz could indicate the presence of relaxation peak at higher frequencies than 1 MHz.

Apparently, there are clear effect of doping towards $\varepsilon^{{\prime }}$ values from low to higher frequencies with heavy, medium and light–sized electrical dipoles contributed in $\varepsilon^{{\prime }}$ response at low, medium, and high frequencies, respectively. The $\varepsilon^{{\prime }}$ values keep decreased as frequencies increased, and $\varepsilon^{{\prime }}$ values between all compounds are slightly differ from each other, most likely due to small difference of density between the compounds. The highest density of Mn 0.06 could be the reason of its highest value of $\varepsilon^{{\prime }}$ from 50 Hz to 650 kHz compared to the other compounds^19^. Nonetheless, the lowest decrease of $\varepsilon^{{\prime }}$ variation in Mn 0.08 in medium frequencies range and its highest $\varepsilon^{{\prime }}$ value at 1 MHz compared to other compounds can be pertained for having larger medium-sized dipoles with higher abundance, which is related to smallest *Tan δ* in the compound at frequencies above 400 kHz as in inset of Fig. 6b. The variation of $\varepsilon^{{\prime }}$ response can be explained in terms of its interrelation with average grain size in inset of Fig. 6a. Larger grains size can form larger medium-sized dipoles and probability of higher amount of medium-sized dipoles. Thus, production of larger electrical dipole moments with larger polarization effect could took place. This suit to explain the relationship between variation in $\varepsilon^{{\prime }}$ in medium frequencies and average grain size. This factor might be the reason of Mn 0.08 possessed highest $\varepsilon^{{\prime }}$ at 750 kHz and above. Other reports that were related to this interrelation between $\varepsilon^{{\prime }}$ and grain size has been recorded before^49^. In comparison to the pristine SrLaLiTeO_6_^43^, there is enhancement of $\varepsilon^{{\prime }}$ values in all Mn^6+^ doped SrLaLiTeO_6_ compounds. From the Eq. (8) below:

8 $$C = \varepsilon_{o} \varepsilon^{{\prime }} \left( {{\raise0.7ex\hbox{$A$} \!\mathord{\left/ {\vphantom {A d}}\right.\kern-\nulldelimiterspace} \!\lower0.7ex\hbox{$d$}}} \right)$$

where $\varepsilon_{o}$ is the vacuum permittivity, *A* is the area of contact between compounds and electrode, and *d* is thickness of compounds, the calculated values of capacitance at 1 MHz in room temperature for Mn 0.02, Mn 0.04, Mn 0.06, Mn 0.08, and Mn 0.10 were 7.77 nF, 9.4 nF, 9.15 nF, 11.2 nF, and 9.7 nF, respectively. These results clearly showed the highest capacitance obtained by Mn 0.08 at 1 MHz, in accordance with the $\varepsilon^{{\prime }}$ trend.

**Figure 7:**
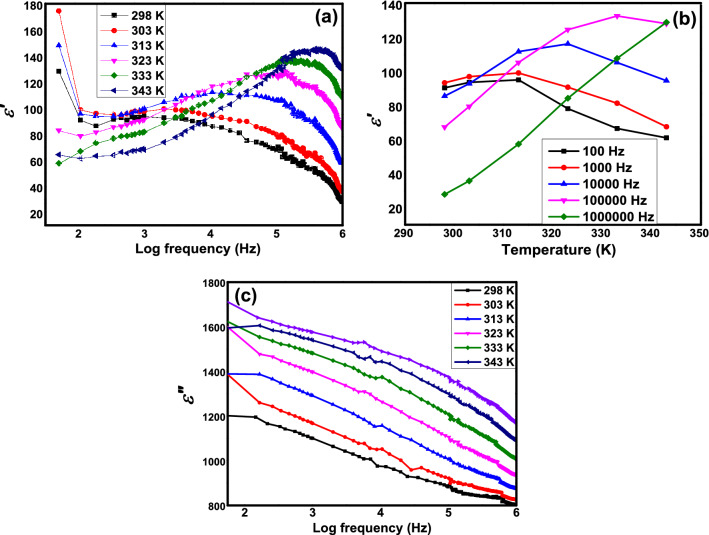
(**a**) Variation of $\varepsilon^{{\prime }}$ in SrLaLiTe_1−*x*_Mn_*x*_O_6_ (*x* = 0.08) with respect to temperatures, (**b**) variation of $\varepsilon^{{\prime }}$ in SrLaLiTe_1−*x*_Mn_*x*_O_6_ (*x* = 0.08) with respect to frequencies, and (**c**) variation of $\varepsilon^{{\prime \prime }}$ in SrLaLiTe_1−*x*_Mn_*x*_O_6_ (*x* = 0.08) with respect to temperatures.

Figure 7a illustrates variation of $\varepsilon^{{\prime }}$ value as function of temperature in the range of 298 K to 343 K of Mn 0.08 sample. All other samples exhibit almost identical trend of plot as in Mn 0.08 (not shown). At minimum frequency, the $\varepsilon^{{\prime }}$ values for each temperature enhanced as temperature elevated from 298 to 313 K. In higher temperatures from 323 to 343 K, the values of $\varepsilon^{{\prime }}$ at minimum frequency dropped. Meanwhile, $\varepsilon^{{\prime }}$ variation with respect to frequency in 298 K exhibit steady decreasing trend at medium and high frequencies. Generally, values of $\varepsilon^{{\prime }}$ enhanced in these frequency ranges as temperatures elevated. Figure 7b illustrates the variation of $\varepsilon^{{\prime }}$ against temperature for sample of Mn 0.08 at selected frequencies. Apparently, there were more dispersion of $\varepsilon^{{\prime }}$ with temperature as temperature elevated. All frequencies, except at 1 MHz, exhibited non-linearly dependence of $\varepsilon^{{\prime }}$ response against temperature. For each frequency, the decrease of $\varepsilon^{{\prime }}$ value occurred in different temperatures. Each decrease shifted towards the higher temperature as frequencies increased. On the other hand, the graph increased almost linearly with temperatures at 1 MHz.

The small $\varepsilon^{{\prime }}$ values in low frequencies range in 323–343 K in Fig. 7a can be interpretated as inability of electrical dipoles to follow the external field efficiently which most probably due to the scattering of the dipoles when external heat energy added. Thermal activation started to show effect at 1 kHz and increase the $\varepsilon^{{\prime }}$ values where the medium and light dipoles were able to cope with external field ascribed to external heat energy which aided the electrical dipoles to follow external field. At frequencies higher than 100 kHz, these polarizations failed to effectively follow the external electrical field. The increasing trends and enhanced values of $\varepsilon^{{\prime }}$ from 100 Hz to 100 kHz, in temperatures of 323–343 K as in Fig. 7a,b could indicated the dielectric resonant phenomenon in the compounds. Reports have discussed the resonance occurrence and related to the damped harmonic oscillator model^50–52^. However, there are no $\varepsilon^{{\prime \prime }}$ peaks in Fig. 7c were available at the same frequencies range to support this argument. Thus, the probability of resonance occurrence can be dismissed.

**Figure 8:**
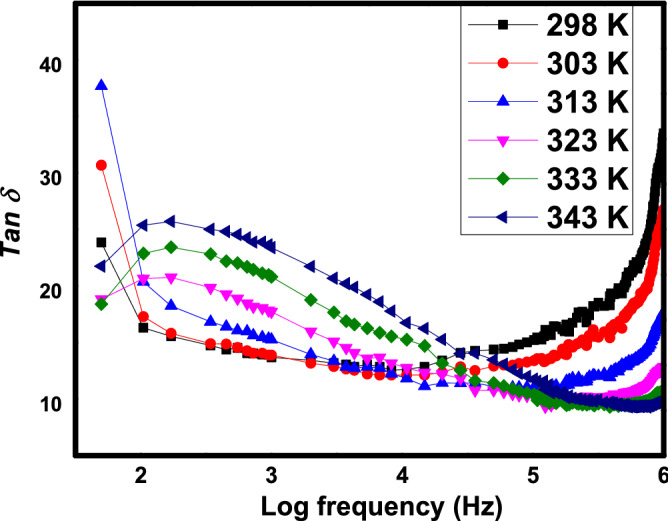
Tan δ of SrLaLiTe_1−*x*_Mn_*x*_O_6_ (*x* = 0.08) at different temperatures.

Figure 8 exhibits changes of Tan δ variation in Mn 0.08 against various temperature from 298 to 343 K. All other samples exhibited almost same pattern of Tan δ as in Mn 0.08 (not shown). The presence of two peaks at minimum and maximum frequencies were apparent in almost all temperatures. As temperature elevated until 313 K, the minimum frequency peaks are enhanced meanwhile maximum frequency peaks are reduced. In temperature of 323–343 K, there were absence of the minimum frequency peaks. Meanwhile, there is a possibility of maximum frequency peaks to shift towards higher frequencies as temperature elevated.

The presence of peaks at minimum frequency in temperature of 298–313 K in Fig. 8 could be because of heavy electrical dipoles cannot effectively follow the alternation of electric field. In higher temperatures from 323 K until 343 K, smaller values of Tan δ at minimum frequency corresponded to the small value of ${\varvec{\upvarepsilon}}$’ at the same frequency. On the other hand, the presence of peaks at 1 MHz frequency and the possibilities of the peaks to shift towards the higher frequencies as temperature elevated can be interpreted as the possibility of relaxation peaks presence^53,54^ at frequencies higher than 1 MHz since the electrical dipoles unable to match the alternation of higher external field frequencies.

The obtained results from our study showed that small B–site doping sample preparation can significantly alter the dielectric properties of tellurium based double perovskite. In addition, the $\varepsilon^{{\prime }}$ values at 1 MHz and in room temperature are comparable or better results compared to some reports with full or partial A–site doping samples preparations. Table 4 summarized the comparison of our results with the results from other reports.

**Table 4:** The highest values of $\varepsilon^{{\prime }}$ at 1 MHz from our study in comparison with results from other reports.

Result from this study		Results from other reports		
Compound	Obtained $\varepsilon^{{\prime }}$ at 1 MHz	Compounds	Obtained $\varepsilon^{{\prime }}$ at 1 MHz	References
SrLaLiTe_0.92_Mn_0.08_O_6_	31.5	BaBiNaTeO_6_	33.2	^18^
		BaLaNaTeO_6_	12.9	
		BaBiLiTeO_6_	38.0	^19^
		SrBiLiTeO_6_	30.0	
		SrLaLiTeO_6_	14.2	^43^
		SrLa_0.75_Nd_0.25_LiTeO_6_	33.1	
		SrLa_0.25_Nd_0.75_LiTeO_6_	34.5	
		SrNdLiTeO_6_	19.8	

## Conclusion

SrLaLiTe_1−*x*_Mn_*x*_O_6_ double perovskites was successfully synthesized by applying solid state reaction method. All samples were confirmed to crystallized in monoclinic symmetry with *P2*_*1*_*/n* space group. The existence of maxima at specific wavenumbers confirms the formation of Te^6+^/Mn^6+^–O–Li^+^ octahedral structure. The grain size of the compounds gets larger towards higher concentration doping until *x* = 0.08. The reduction of optical band gap can be deduced because of the doping of Mn^6+^ cations that capable to reduce the gap distance between highest occupied molecular orbital (HOMO) and lowest unoccupied molecular orbital (LUMO) in the compound. Nevertheless, all compounds showed absorption within visible light range which make the application for compounds in optoelectronic device such as solar cell can be realized with modifications on its electrical conductivity or tuning its *E*_*opt*_. Highest dielectric real permittivity and lowest dielectric losses at 1 MHz was recorded by Mn 0.08. This behaviour can be elucidated on the basis of grain size. This study shows that the dielectric and optical characteristics of tellurium based double perovskite can be tailored through small B–doped doping method. Further studies related to annealing temperature, full doped sample preparation or other means can be done to increase the value of dielectric real permittivity in the future. The enhancement in dielectric and optical characteristics of the studied compounds revealed good potential for the materials to be used in electronic applications.
